# Unpredictive linguistic verbal cues accelerate congruent visual targets into awareness in a breaking continuous flash suppression paradigm

**DOI:** 10.3758/s13414-021-02297-y

**Published:** 2021-03-30

**Authors:** Chris L. E. Paffen, Andre Sahakian, Marijn E. Struiksma, Stefan Van der Stigchel

**Affiliations:** 1 Department of Experimental Psychology & Helmholtz Institute, Utrecht University, Heidelberglaan 2, 3584 CS Utrecht, the Netherlands; 2 Department of Language, Literature & Communication, Utrecht Institute of Linguistics OTS, Utrecht University, Utrecht, the Netherlands

**Keywords:** Embodied cognition, Visual awareness, Language

## Abstract

One of the most influential ideas within the domain of cognition is that of embodied cognition, in which the experienced world is the result of an interplay between an organism’s physiology, sensorimotor system, and its environment. An aspect of this idea is that linguistic information activates sensory representations automatically. For example, hearing the word ‘red’ would automatically activate sensory representations of this color. But does linguistic information prioritize access to awareness of congruent visual information? Here, we show that linguistic verbal cues accelerate matching visual targets into awareness by using a breaking continuous flash suppression paradigm. In a speeded reaction time task, observers heard spoken color labels (e.g., red) followed by colored targets that were either congruent (red), incongruent (green), or neutral (a neutral noncolor word) with respect to the labels. Importantly, and in contrast to previous studies investigating a similar question, the incidence of congruent trials was not higher than that of incongruent trials. Our results show that RTs were selectively shortened for congruent verbal–visual pairings, and that this shortening occurred over a wide range of cue–target intervals. We suggest that linguistic verbal information preactivates sensory representations, so that hearing the word ‘red’ preactivates (visual) sensory information internally.

**Supplementary Information:**

The online version contains supplementary material available at 10.3758/s13414-021-02297-y.

According to the idea of embodied cognition, the experienced world is the result of an interplay between an organism’s (neuro-)physiology, its sensorimotor system, and its environment (Barsalou, 1999, 2008; Varela et al., 1991). A concrete example of this theory is that hearing the word ‘red’ activates this word’s sensory representations—in this case, its color. In recent years, many studies have, in support of the embodied cognition account, reported on the ability of language to affect visual perception. For example, it has been shown that linguistic labels can (1) enlarge perceived differences between targets and distractors in visual search (Lupyan, 2008; Lupyan & Spivey, 2010; Lupyan & Swingley, 2018), (2) affect visual motion perception (Dils & Boroditskty, 2010; Francken et al., 2015; Meteyard et al., 2007), (3) affect contrast sensitivity (Pelekanos & Moutoussis, 2012), and (4) affect face perception (Landau et al., 2010). Moreover, the influence of communicative acts (i.e., language) on vision appears not to be limited to the human species, as Suzuki (2018) showed that vision of Japanese tits (*Parus minor*) is affected by alarm calls of fellow birds: When hearing such calls, Suzuki’s birds became more perceptive to objects resembling snakes. Although it is disputed whether the above results actually show that cognition is embodied (see, for example, Firestone & Scholl, 2016), the emerging conclusion is that linguistic labels activate neural structures also involved in actually perceiving the information the labels refer to. Evidence for the latter is provided by studies showing that linguistic labels (e.g., the word ‘red’) activate neural structures also involved in processing sensory information (e.g., the color red; Chao & Martin, 1999; Goldberg et al., 2006; Kellenbach et al., 2001; Martin et al., 1995; Oliver & Thompson-Schill, 2003; Simmons et al., 2007).

A possible functionality of linguistic labels activating sensory representations is that this might speed up conscious access of relevant sensory information. That is, linguistic labels enable an organism to respond faster to matching rather than mismatching information because the information’s access to consciousness is sped up. One way to address this adaptive value of embodied cognition is to assess the speed by which information is available for conscious report. The recently developed paradigm breaking continuous flash suppression (b-CFS; a variant of continuous flash suppression; Tsuchiya & Koch, 2005) provides a means to operationalize this effort. In this paradigm, a target presented to one of the two eyes is gradually increased in intensity while a high-contrast flickering mask is presented to the other eye (see Fig. 1; for a recent review, see Moors et al., 2017). Phenomenologically, this results in the target being initially suppressed from awareness, while suddenly entering awareness when a sufficient intensity is reached. Examples of prioritized access using this method are the advantage of (1) upright over inverted faces (Gray et al., 2013; Jiang et al., 2007; Zhou et al., 2010), (2) information matching rather than mismatching the content of working memory (Ding et al., 2019; Gayet et al., 2013; Gayet, van Maanen, et al., 2016; van Moorselaar et al., 2017), and (3) information signaling threat (Gayet, Paffen, et al., 2016).

**Fig. 1:**
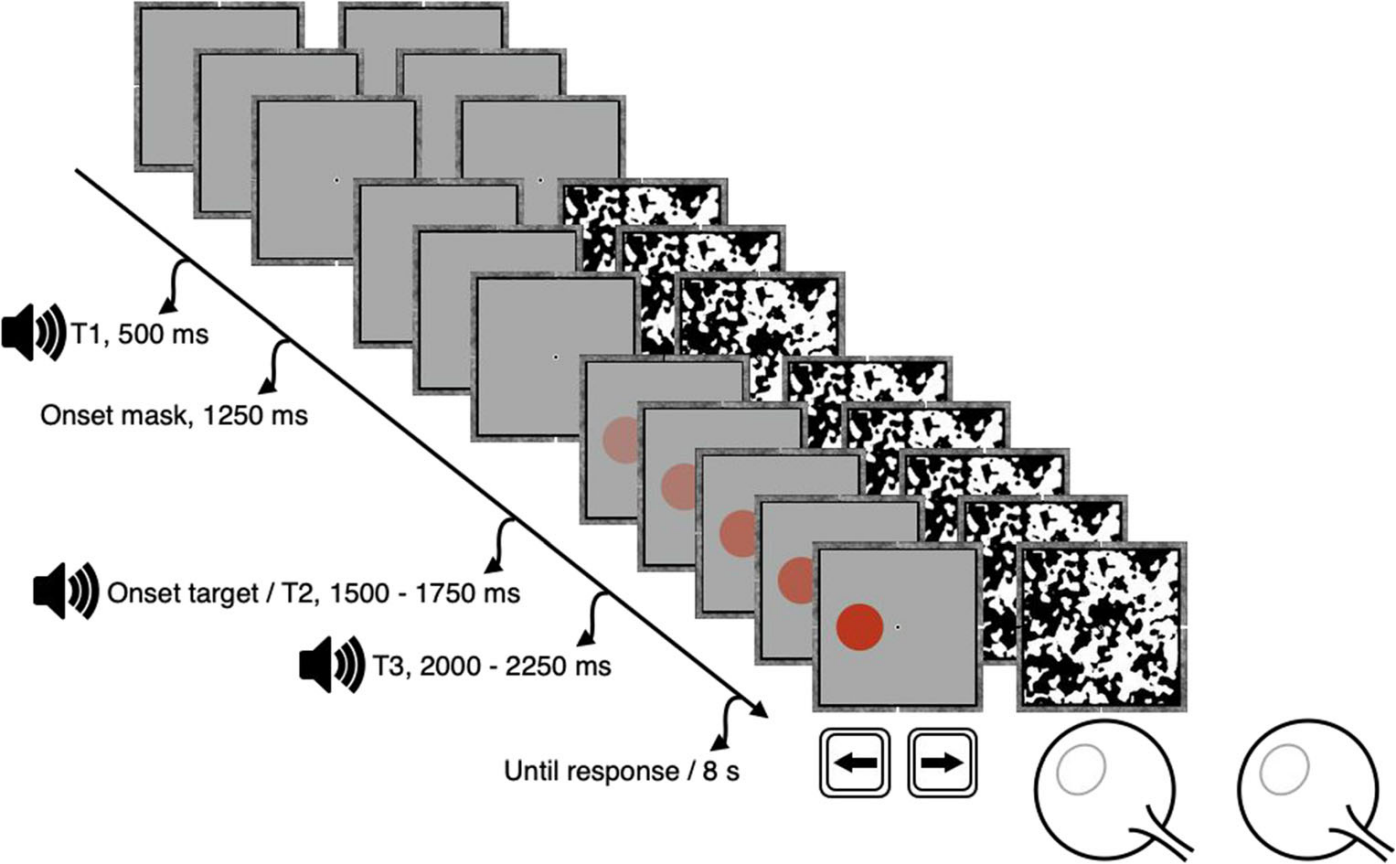
The sequence of events during a single trial. In short, an observer viewed the display through a mirror stereoscope, then, 1,250 ms after starting a trial, the dynamic mask was presented to one of the eyes; between 250 and 500 ms later, a target disc started to gradually increase in opacity (ending after 1 s). A spoken word indicating a color label was presented 500 ms, 1,500–1,750 ms, or 2,000–2,250 ms after the start of the trial. The observer was instructed to indicate whether the target disc appeared left or right of the fixation dot in a choice reaction-time task

Does linguistic information similarly speed up access to consciousness of matching visual information? This question was addressed by Lupyan and Ward (2013), and more recently by Forder et al. (2016), and Ostarek and Huettig (2017). In Lupyan and Ward’s study, participants were required to detect the presence of targets that were preceded by either congruent or incongruent linguistic verbal labels (e.g., a picture of a zebra preceded by the word ‘zebra’), and were simultaneously masked by continuous flash suppression.^1^ Their results show that (a) sensitivity (objectified by *d'*) for targets was higher for congruent compared with incongruent labels, and (b) reaction times to congruent targets were decreased compared with incongruent targets. These results are similar to those by Forder et al. (2016), who found, using *b*-CFS, similar results for participants detecting colored patches preceded by verbal color labels. Important for the interpretation of the results of both studies is that, when a target was present, it was in 80% of the cases preceded by a congruent cue. This means that even though participants could not use the cue to predict whether an object was present or not (the ratio present–absent trials was 1:1), they could use a simple rule during the experiment: When a target is present, it will in most cases be congruent with the verbal label (specifically: in 50% of the trails, no target would be present, in 40% a congruent target, and in 10% an incongruent target). Because of this rule, it made sense for participants to hold the verbal label in their working memory so as to keep the information active until a target was (potentially) presented. In 90% of the trials, this rule would not work against a participant: either no or a congruent target would be presented. Only in 10% of the trials, applying the rule would be ineffective: The target would turn out to be incongruent with the label. In that sense, the experiments of these studies potentially addressed, rather than language’s ability to affect visual perception, whether holding information in working memory prioritizes access to visual information, a question that has been addressed and answered (that is: confirmed) by several previous studies (Ding et al., 2019; Gayet et al., 2013; Gayet, van Maanen, et al., 2016; van Moorselaar et al., 2017) Lupyan and Ward (2013) *did* perform an experiment in which the above rule did not hold: in their Experiment 3, targets that varied between forming a circle or a square were preceded by the verbal labels ‘circle’ or ‘square,’ or by auditory noise (as a baseline condition). As the chance of encountering either a circle or square target did not hinge on the identity of the cue in this experiment, uploading the cue to working memory would be ineffective in this experiment. Although increased sensitivity for congruent couplings was observed in this experiment as well, reaction times (addressing accelerated access to awareness) did not differ between congruent and incongruent verbal–visual pairs. Ostarek and Huettig (2017), applying a method similar to that of Forder et al. (2016)—simultaneous presentation of CFS and a target—most recently performed two experiments with the same incidence of congruent and incongruent trials. In addition, they varied the cue–target asynchrony in their study. The results showed that performance for detecting congruent targets was higher than for incongruent targets, but not for all cue–target intervals: increased sensitivity (objectified by *d'*) was not observed for cues presented 200 ms after occurrence of the target, nor for targets preceded by the cue by 600 ms. Increased sensitivity *was* observed for cues and targets with simultaneous onset and for cues preceding the target by 200 ms.

To summarize, three studies have so far investigated access to awareness of visual targets following linguistic verbal labels. Two studies (Lupyan & Ward, 2013; Ostarek & Huettig, 2017), did *not* investigate whether congruent visual targets are entering visual awareness faster. Rather, the experiments were targeted at the ability to detect briefly presented targets that were being masked by continuous flash suppression. This notion is important, as our primary goal here is to investigate prioritized access, assessed by measuring *the time it takes* for targets to enter visual awareness. As has been noted by Stein (2019), combining a signal detection approach with a speeded reaction-time task (as these two previous studies did) is not ideal, as performing a speeded task will affect perceptual sensitivity measures.^2^ When assessing the time it takes for targets to enter visual awareness, *breaking* continuous flash suppression (b-CFS) provides a unique tool (Stein, Hebart & Sterzer, 2011).^3^ Importantly, even though CFS and b-CFS might, at first sight, appear as similar methods, it is important to note that presenting target and mask simultaneously (as in the former method) leads to results that can differ both quantitatively *and* qualitatively from results obtained by gradually introducing the target (as in the latter method; Kaunitz et al., 2014; Stein, 2019). In the one study in which b-CFS *was* applied to assess the time it takes for visual targets to enter visual awareness following linguistic verbal labels (i.e., that of Forder et al., 2016), the incidence of congruent trials was much higher than the incidence of incongruent trials (potentially introducing the added value of holding information in working memory, as discussed above). Thus, as it is still not known whether targets congruent rather than incongruent to linguistic verbal cues are prioritized for entering visual awareness without the potential confound of a role of working memory, we here apply b-CFS in two experiments in which the incidence of congruent trials was not higher than that of incongruent trials. In fact, to make the recruitment of working memory especially unlikely, incongruent trials outnumbered congruent trials in our experiments. Moreover, we investigate whether access is prioritized for congruent visual targets, and/ or delayed for incongruent visual targets. Although this issue too was addressed by Forder et al., it is important to note that in that study auditory noise burst were used. The disadvantage of using noise bursts is that, although being neither congruent or incongruent with a target image, they are also not words, thereby lacking (linguistic) meaning, and have totally different spectral qualities as spoken words have. For these reasons we here include words being neither congruent nor incongruent with target images presented during b-CFS to contrast the congruent and incongruent conditions against. In addition, we investigate the timing of these verbal-visual interactions, by varying the cue–target stimulus onset asynchrony, which was not addressed by Forder et al. (2016). 

## Experiment 1

### Method

#### Observers

The number of observers was based on a Bayesian stopping rule (see below). We collected data from 22 observers of which we excluded 4 from further analysis (further explained in the ‘Results’ section). We analyzed the data of 18 observers (3 males; average age 23.06, SD = 4.61). All observers signed a written consent form before participating in the experiment and received a monetary reward upon completion of the experiment. The experiments were conducted according to the declaration of Helsinki and were approved by the local ethics committee. All observers reported to have (corrected-to) normal vision, normal hearing, and being native Dutch speakers. All were tested for color-blindness with the Ishihara test plates, and for stereoscopic vision with the TNO test for stereoscopic vision (Ishihara, 1918; Walraven, 1971).

#### Apparatus and stimuli

Observers viewed the monitor through a mirror setup which allowed for simultaneous presentation of two half-images to each eye separately. The effective viewing distance from the monitor to the observer’s eyes was 61 cm. A linearized 27-inch Asus PG279Q LCD monitor (2,560 × 1,440 pixels, 144-Hz refresh rate) connected to a PC (Windows 10) was used to display the visual stimuli. Headphones (Sennheiser HD 201, pitch range: 21 Hz–18 kHz), connected to a soundcard (Sound Blaster Audigy Fx) were used to deliver the auditory cues with the volume setting on 50% of the maximum. The presentation of the stimuli, as well as response collection, was coded and handled using Psychophysics Toolbox Version 3 (Brainard, 1997; Pelli, 1997) and MATLAB 2016a.

The auditory cues consisted of the spoken Dutch words ‘rood,’ ‘groen,’ and ‘blauw’ (‘red,’ ‘green,’ and ‘blue,’ respectively). All words were recorded in a single session with a 26-year-old female native Dutch speaker, in a recording booth, using the Audacity software. All auditory cues were adjusted in duration (a total duration of 300 ms), pitch (median: 215 Hz, variation 25%) and intensity to match each other as much as possible. The editing of the recorded sound files was done using the PRAAT software and the Vocal Toolkit plugin (Boersma & van Heuven, 2001).

To realize binocular fusion of the visual stimuli, two identical Brownian noise (i.e., 1/f^2^) square frames with a height and width of 7.5 deg visual angle (VA) and a thickness of 0.4 deg VA were used. All visual stimuli (the noise masks, visual targets, fixation point, and also the instructional text) were presented within the bounds of these frames. Saturated red, green and blue colored circles with a diameter of 1.2 deg VA were used as targets in the b-CFS task. The luminance for the red and green targets, as well as that of the grey background, was subjectively matched to the luminance of the blue target by means of heterochromatic flicker photometry (Kaiser & Comerford, 1975; Wagner & Boynton, 1972). The alpha value (i.e., opacity) of each color was limited (red: 50%, green: 80%, blue: 30%; obtained in a pilot study) in order to obtain response times that were comparable for the colors. The targets were presented at a fixed distance of 1.8 deg VA from the center of the fixation point, on a random position within π/4 radians above or below the horizontal midline. The fixation point was a white circle with a diameter of 0.2 deg VA with a smaller black circle with a diameter of 0.1 deg VA inside it and was always present in the center of both square frames. The masks used to achieve continuous flash suppression were created by filtering pink (1/f) noise using a Gaussian low-pass filter (sigma = 7.5 deg VA) and making the resulting image binary (black and white, >99% Michelson contrast). For each observer, a set of 200 new masks was created before the experiment. In each trial, these 200 masks were presented in a random order for 100 ms per mask (i.e., the masks changed at a rate of 10 Hz).

#### Procedure

Before starting the experiment, observers adjusted the position of the square fusion frames on the screen until they could comfortably perceive a single fused square frame. They did this while fixating the fixation point, which they were asked to do throughout the entire experiment. Since it was recently reported that eye dominance is dependent on the task with which it is determined (Ding et al., 2018), observers started out with performing a simple b-CFS task. In 30 trials, the dynamic mask was presented to one eye, while a target was presented to the other. In half of the trials, the mask was presented to the left and the target to the right eye; in the other 15 trials, the other way round, while the order of the trials was shuffled. The configuration (e.g., mask left; target right) which resulted in the slowest average response time determined the presentation of mask and target in the main experiment; the dominant eye was operationalized as the one to which mask presentation lead to the longest RTs.

The main experiment consisted of 324 experimental trials (illustrated in Fig. 1) in six blocks of equal length, with a prompt to take a break between each block. The observer initiated each trail by a space press. After 1,250 ms from the start, CFS was initiated, and after another delay of 250 to 500 ms (a randomly selected multiple of 50 ms, between 250 and 500 ms), the visual target was introduced by linearly increasing the alpha value from zero to its maximum value within 1,000 ms, after which it remained present until a response was given. The target was presented to either the left or the right of the fixation point. The auditory linguistic cue could be presented (i.e., the onset of the sound file) at three different time points: at 500 ms after the start of a trial (T1); simultaneously with the introduction of the visual target (T2); or 500 ms after the introduction of the visual target (T3). This implies that the cue–target stimulus onset asynchrony (hereafter: SOA) in this experiment was 1,000–1,250 (T1), 0 (T2), and −500 ms (T3) respectively. A trial would end when a response was given or when no response was given within 8 seconds after the target was introduced. Observers responded by pressing down the left or right arrow keys with their right hand and pressed the space bar with their left hand.

Trials were deemed invalid whenever observers responded too soon (i.e., the target had not appeared yet), erroneously (i.e., responded left when the target was presented to the right or vice versa), or too late (i.e., more than 8 seconds after introduction of the target). Invalid trials were added at the end of the experimental trials to be redone. An observer would be removed from analysis when performing 40 or more invalid trials.

We used a full factorial design (3 cue timings × 3 target colors × 3 cue colors × 2 target locations), which resulted in 54 unique combinations of conditions. Each unique combination was presented six times, which amounted to the total number of 324 experimental trials. Eye-dominance was not a factor, because it was the same in all experimental trials. The trial order was randomized for each observer.

#### Bayesian stopping rule

Because all analyses were done using Bayesian statistics, we kept including observers until reaching a Bayes factor of 10 or greater, or of 1/10 or smaller, for the hypothesis that congruent sounds led to shorter RTs than incongruent sounds. That is, after a participant finished the experiment, we checked the data set using the exclusion rule explained above (more than 40 invalid trials). If not excluded, we calculated the Bayes factor until reaching the one needed for the stopping rule. One of the advantages of using Bayesian statistics is that evidence for the null hypothesis can also be quantified (in our case, a Bayes factor of 10). We calculated the Bayes factor in JASP using a one directional Bayesian T-test, using the standard Cauchy prior width of (√2)^−1^ ≈ 0.707 (JASP Team, 2020). We dubbed the one directional test (i.e., RTs following congruent cues are faster than RTs following incongruent cues) justifiable since the congruency effect was expected based on the work of Forder et al. (2016), Ostarek and Huettig (2017), and Lupyan and Ward (2013). The results of this approach can be observed in Supplementary Figure S1.

#### Data analysis

The choice of the pipeline to analyze b-CFS results has been shown to dramatically affect the outcome of an experiment (Kerr et al., 2017). For example, Gayet and Stein (2017) have shown that some of the variance in response times between different conditions in a b-CSF paradigm is explained by individual differences in absolute response times. Because we are only concerned with the variance induced by congruency and not by individual differences, we eliminated this unwanted variance by latency-normalizing the response times. Importantly, this procedure increases sensitivity to measure the effect of interest (Gayet & Stein, 2017). This method has the additional advantage of producing a distribution of response times that approach a normal distribution (much more so than raw RTs). We implemented Gayet and Stein’s (2017) method as follows: For each observer we first defined an overall response time by calculating the average of the median raw response times of all conditions. Next, the normalized response time for a given condition was calculated by dividing its raw median response time by the overall response time.

### Results

The number of observers needed to reach the threshold of our stopping rule (see Method) was 18. This number was reached after two observers were removed from the analysis because they performed 40 or more trials invalidly and two additional observers were removed because of (1) the inability to fuse the half-images throughout the experiment, and (2) failing to comply with the task instructions. The average of the median response times of all trials of all included observers was 1,359 ms (*SD* = 245 ms).

**Fig. 2:**
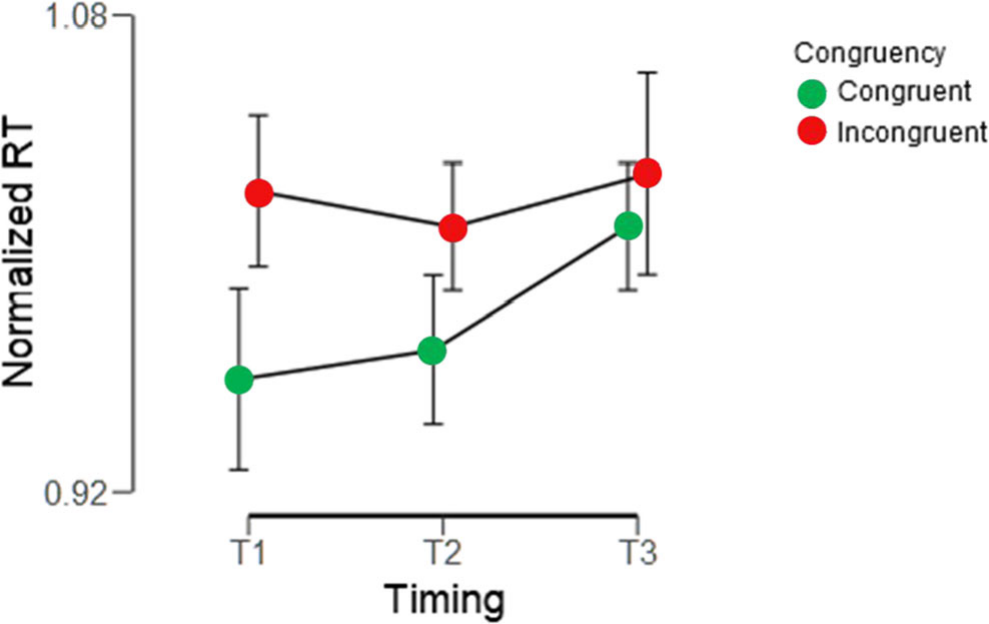
Results of Experiment 1. Normalized (see text) reaction times where plotted against the timing of auditory congruent (red dots) and incongruent (green dots) verbal linguistic labels. Error bars represent 95% confidence intervals. (Color figure online)

Results are displayed in Fig. 2. The hypothesis that congruent trials would result in faster RTs than incongruent trials was supported by a BF_10_ of 13.8. In terms of raw response times, observers were on average 4.21% (*SD* = 5.70 %) faster on congruent trials compared with incongruent trials. In terms of actual duration, RTs of congruent trials were on average 69 ms (*SD* = 99 ms) faster than RTs of incongruent trials. Individual data can be inspected in Supplementary Figs. S1 and S2.

To evaluate the effect of the timing of the cue, we performed a 2 (congruent, incongruent) × 3 (T1, T2, T3) Bayesian repeated-measures analysis of variance (ANOVA). To determine how likely it is that we observe our data given that there is an interaction between congruency and timing, we need to divide the BF_10_ of “Congruency + Timing + Congruency × Timing” by the BF_10_ of “Congruency + Timing.” That would give us the BF_10_ (i.e., evidence in favor) of only the interaction Congruency × Timing. Performing this final calculation gives us a BF_10_ = 302.6 / 458.6 = 0.66, which amounts to barely worth mentioning in Bayesian terms.

## Experiment 2

The results of Experiment 1 clearly show that verbal linguistic labels speeded access to awareness of congruent target colors. In addition, the results show that this occurred over a wide range of SOAs: the congruency effect occurred irrespective of the latency between cue and target. What we cannot know, however, is whether the congruency effect was caused by a facilitatory effect of congruent cues (leading to faster access to awareness), an inhibitory effect of incongruent cues (leading to slower access to awareness), or both. In a second experiment, we added a third condition to specifically tease apart these three options. This third condition included verbal linguistic labels not related to color (i.e., “five”). If congruent labels speed up conscious access, RTs in the neutral condition should be longer than those of the congruent condition; if incongruent labels slow down conscious access, RTs in the neutral condition should be shorter than those of the incongruent condition. In addition, the second experiment allowed us to find additional, replicatory evidence for the conclusion that verbal linguistic cues accelerate access to awareness of congruent visual targets.

### Method

#### Observers

Nineteen observers participated in this experiment, of which one was excluded from further analysis because of a large amount of incorrect trials. The data of the remaining 18 observers (seven males; average age = 22.67 years, *SD* = 3.02) was analyzed. Two of the observers had also taken part in Experiment 1, but were naïve as to the goal of the current study. Requirements for inclusion as well as consent and approval was the same as for Experiment 1.

#### Apparatus and stimuli

The apparatus and stimuli were identical to those of Experiment 1 with the addition of three auditory linguistic cues: the Dutch words “Jan” (a Dutch male name), “kant” (‘side’ or ‘lace’), and “vijf” (‘five’) were added to the three color labels used in Experiment 1. These labels were obtained from the same speaker and in the same recording session of the cues of Experiment 1. Also, these cues were adjusted using the same procedure as the cues from Experiment 1.

#### Procedure

The procedure was the same as that of Experiment 1, except for the fact that the third SOA condition (T3) was left out (since different SOAs did not differentially affect the RTs in Experiment 1), and that this experiment consisted of 360 trials in six blocks of equal length (there were now 72 unique combinations of conditions). We held onto two SOAs instead of one in this experiment to avoid a scenario in which observers would start anticipating the target based on a limited range of cue target SOAs.

#### Stopping rule and data analysis

Since our experiments were designed to find evidence for accelerated access to awareness, the main hypothesis for Experiment 2 was that the neutral condition would be different from the congruent condition, and our secondary hypothesis was that the neutral condition would *not* be different from the incongruent condition. This hypothesis is also in line with the results of Experiment 2 of Forder et al. (2016), for which they reported that (1) congruent verbal color labels lead to shorter RTs than the neutral condition, and (2) incongruent verbal labels did not lead to longer RTs than their neutral condition. We therefore decided to test observers until we reached a Bayes factor of 6 in favor for our primary hypothesis (or a Bayes factor smaller than 1/6, which would be the similar strength of evidence in favor of the null hypothesis). We chose the Bayes factor of 6 (instead of 10 in Experiment 1), as the stopping rule of Experiment 1 was based on a one-tailed rule (congruent sounds led to *shorter* RTs than incongruent sounds), whereas the stopping rule of Experiment 2 was based on a two-tailed rule (the neutral condition would be different from the congruent condition). Importantly, and in addition to this stopping rule, we decided to test a minimum of 18 observers (the same number as in Experiment 1) to obtain, at least, similar strength in evidence. We again calculated the Bayes factors in JASP using a Bayesian RM ANOVA and performing post hoc tests on congruency (where we used the standard Cauchy prior width of (√2)^−1^) (JASP Team, 2020). As for the results of Experiment 2, statistical analyses were again performed on latency-normalized response times. In the analysis, we collapsed the data of the T1 and T2 SOA conditions, since we learned from Experiment 1 that different SOAs did not affect RTs differentially.

### Results

**Fig. 3:**
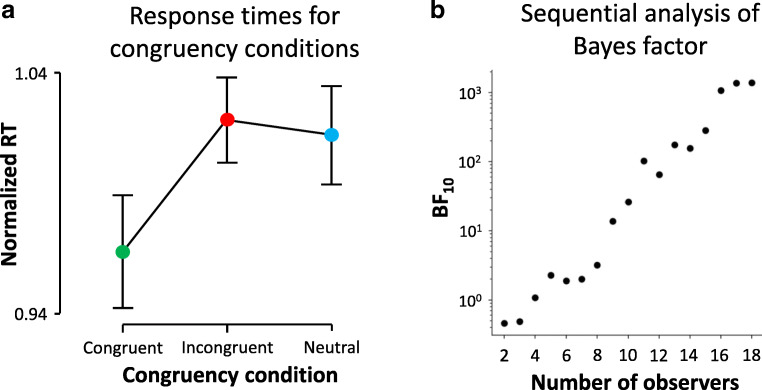
Results of Experiment 2. **a** Normalized RTs for the condition where the verbal linguistic label was (1) congruent with the color of the visual target (green), incongruent with the color of the visual target (red), or (3) neutral with respect to the color of the visual target. Error bars represent 95% confidence intervals. **b** Sequential Bayes factors (BF10 plotted on a log-scale, as a function of number of observers). (Color figure online)

The performance for indicating the side of presentation of the target was 98.3% (*SD* 1.9%). The average of the median response times of all trials of all included observers was 1,339 ms (*SD* = 202 ms), which is comparable to Experiment 1. Again, we find strong evidence for an effect of congruence: the main effect reveals a BF_10_ of 1,357 (Fig. 3). Post hoc testing revealed a BF_10_ of 32.9 for the contrast between the congruent and incongruent condition, and a BF_10_ of 7.7 for the contrast between the congruent and the neutral condition. The latter result is important because this is where the stopping rule was based on. Finally, the contrast between the incongruent and neutral condition revealed a BF_10_ of 0.28, indicating evidence for the null hypothesis. From these results, it can be concluded that congruent verbal linguistic labels accelerated access to awareness of colored targets; we find no evidence for an inhibitory effect of incongruent verbal linguistic labels.

## General discussion

In two experiments we evaluated the hypothesis that verbal linguistic labels accelerate access to awareness of congruent visual targets. Both experiments confirmed the main hypothesis: Access to awareness of congruent targets was prioritized compared with that of incongruent targets. Additionally, we investigated the importance of the timing between the presentation of the verbal linguistic label and the visual target, by using three SOAs. The results gave no conclusive evidence that timing mattered: timing did not interact with the effect of congruency. Moreover, Experiment 2 showed that congruent targets were prioritized, whereas incongruent targets were not mitigated to enter awareness.

Within the context of linguistic–visual interactions, our results might be taken (at first sight) as yet another example of the potency of (linguistic) color labels to prime processing of visual targets (see, for example, the studies cited in the Introduction). Within the context of access to awareness, however, our results are not so straightforward. For example, in one of the b-CFS experiments in Gayet et al. (2013), observers were shown a colored probe followed by a target that could be either congruent or incongruent with the probe. In contrast to the other experiments of that study, the observers were instructed to look at the probe passively (instead of holding it active in working memory). The results of this study give no evidence for priming: reaction times to congruent targets were not shorter than those for responding to incongruent targets. In addition, a recent b-CFS study by Gayet, Douw, Van der Burg, Van der Stigchel, and Paffen (2020) presented participants with a written color label (e.g., ‘red’) followed by a congruent or incongruent visual target. Even though the color label induced a measurable priming^4^ effect (secondary targets not suppressed by CFS were responded to faster when congruent), b-CFS reaction times to the visual targets were unaffected by congruency: Congruent targets were not responded to faster than incongruent trials were. Again, presenting colored primes (actual written words here) did not lead to a measurable priming effect. Next to these two studies reporting no evidence for priming-like effects in a b-CFS paradigm, several other studies *do* report evidence for priming. Three of them (Forder et al., 2016; Lupyan & Ward, 2013; Ostarek & Huettig, 2017) were discussed in the Introduction, and we outlined the shortcomings (with respect to the main question of this study) of them. Interestingly, two other studies reporting evidence for priming use paradigms in which targets are *expected* due to the prime. For example, Pinto et al. (2015) and Stein and Peelen (2015) show that *expectation*, for example, based on the identity of the prime, affects b-CFS of visual targets. As we argue in the Introduction and below, we took care in our experiments that participants would not expect a certain target based on the prime (the verbal label): the target was congruent in a minority of trials in our experiments. Two more studies found evidence for semantic priming, one (Costello, Jiang, Baartman, McGlennen, & He, 2009) using written primes and the other (Stein, Thoma, & Sterzer, 2015) using visual objects. All in all, this summary of priming studies reveals that evidence for spoken linguistic labels affecting access to awareness of congruent targets was, up to this point, rather small and our findings not trivial.

It is of relevance to compare our study with that of Forder et al. (2016), Lupyan and Ward (2013) and Ostarek and Huettig (2017), since these studies used a similar paradigm and addressed a similar issue. First and foremost, our two experiments replicate the main conclusions of these studies: verbal linguistic labels affect access to awareness of visual targets which are congruent compared with incongruent with the linguistic labels. In addition, our results extend those of these previous studies: In two out of three experiments conducted by Lupyan and Ward, and in all three conducted by Forder et al., the congruent trials outnumbered incongruent trials. As laid down in the Introduction, this design introduced the possibility of working memory being responsible for speeded access to awareness. In our experiments, the verbal linguistic label was actually incongruent in the majority of the trials (33% congruent, 67% incongruent in Experiment 1; 17% congruent, 33% incongruent in Experiment 2). This design made it very unlikely (in fact, counterproductive) to recruit working memory. Importantly, Lupyan and Ward’s final experiment *did not* use more congruent than incongruent trials. Interestingly, however, their results show that, in this experiment, congruent verbal linguistic labels increased sensitivity (reflected by a higher *d'*), but did not, in contrast to our results, shorten reaction times. The study of Ostarek and Huettig also matched congruent and incongruent trials throughout their experiments, and also found faster RTs following congruent compared with incongruent cues. However, and as will be discussed below, both Lupyan’s and Ostarek’s study used a different method than the one we applied here. Our study is therefore the first to show that access to awareness, assessed by the time it takes to overcome interocular suppression, is prioritized (in terms of shorter RTs) for targets matching congruent verbal linguistic labels without the potential active involvement of working memory, thereby adding support to the hypothesis that verbal linguistic labels activate sensory representations automatically, as has been put forward by Bergen (2012).

As put forward in the Introduction, Ostarek and Huettig (2017) matched the number of congruent and incongruent trials in experiments with a similar goal. It is, however, important to note that this previous study used a different paradigm, as visual targets were presented simultaneously with the onset of the CFS mask. Our question, whether congruent visual targets are propelled into awareness faster than incongruent ones, was operationalized by applying b-CFS. In this method the target is gradually introduced, with some delay, after the onset of CFS. This is important because adopting the previous design (simultaneous onset of target and mask) has been shown to lead to qualitatively different results from the latter (the b-CFS design). For example, high-level processing (e.g., of the emotional expression in a face) is generally disrupted when masked by CFS, while still effective when b-CFS is applied (see Stein & Sterzer, 2014, for a review). One reason for these qualitative differences between CFS and b-CFS is that visual stimulation is different in each. As such, the method of simultaneously presenting target and mask (i.e., CFS) has been classified by Kaunitz et al. (2014) to mimic classic paradigms like dichoptic masking (Macknik et al., 2000) and flash suppression (Wolfe, 1984). Kaunitz et al. (2014) further review evidence for the claim that briefly presented targets (as in Ostarek & Huettig, 2017) are masked primarily by CFS affecting the ‘transient-like’ M-type channels, while prolonged targets with delayed onset (with respect to the mask; as in our study) are masked primarily by CFS affecting the P-type ‘sustained’ channel.^5^ In this regard, our results show that verbal linguistic labels are not only effective in affecting processing of visual targets through affecting transient M-type channels, but also through affecting P-type channels. Another general difference between experiments applying CFS or b-CFS is that they involve a different task: experiments applying CFS are usually nonspeeded, while those applying b-CFS are usually speeded. Returning to the difference between our study and that of Ostarek and Huettig (2017, we also reiterate the observation that combining a signal detection task with a speeded reaction-time task is not ideal (Stein, 2019), as time needed to respond to a target will interact with perceptual sensitivity.

An additional difference between the studies of Lupyan and Ward (2013), and of Forder et al. (2016) on the one side, and that of ours on the other, is that, in their studies, auditory noise bursts were used as baseline to compare against congruent and incongruent conditions (the study by Ostarek & Huettig, 2017) did not include a baseline condition). Because the nature of an auditory noise burst is quite different from a spoken word, we decided to use neutral words, instead.^6^ The advantage of this is that these neutral words match the color labels more closely than noise bursts do (in that they are also words, have meaning and are spoken by the same actor with similar spectral characteristics). Interestingly, the two previous studies report that, on most occasions, RTs to the ‘neutral’ conditions were in between those of the congruent and incongruent conditions. In our study, however, it is clearly the case that RTs of congruent targets were selectively shortened compared with incongruent and neutral targets. In accordance with this result are the findings in the second experiment by Forder et al., for which there was also a selective shortening of RTs for the congruent condition. We reiterate however, that verbal cues predicted the target in the majority of the trials of that experiment.

Another aspect of our endeavor was to investigate the timing of linguistic-visual interactions. Two previous studies that not explicitly investigated the timing of the linguistic–visual interaction used an SOA of 450 ms (Lupyan & Ward, 2013) and 1–2 s (Forder et al., 2016). These studies, together with the present, show that interactions are effective, from long intervals (our study and that of Forder et al., 2016), to short ones (our study and that of Lupyan & Ward, 2013), and even when the verbal linguistic label is presented after onset of the visual target (our SOA of −500 ms). With the risk of becoming repetitive, the wide range of intervals for which the verbal linguistic–visual interactions occurred in our experiments is all the more interesting given the fact that the verbal linguistic labels in our experiments were more often congruent than incongruent. Again, in Lupyan and Ward (2013) and Forder et al. (2016) one could argue that observers held the linguistic labels active (e.g., in working memory) since they would aid detection in case of target present trials (thereby mimicking the influence of working memory on access to awareness, (e.g., Gayet et al., 2013). Of course, it is possible that also in our experiment, observers held the color label active in working memory. We think this to be highly unlikely, however, as holding the label active in working memory would be counterproductive in responding to the target (as incongruent trials outnumbered congruent trials). In fact, the best strategy for participants would be to ignore the labels all together. Ostarek and Huettig (2017) explicitly investigated the timing of linguistic–visual interactions, albeit by, differently from the present study, using simultaneous onset of target and mask. Their results show that, similar to ours, different SOAs were similarly effective in generating a congruency effect: faster RTs were observed for SOAs ranging from +400 (the cue preceding onset of target & mask by 400 ms) to −200 (the onset of the cue following the onset of target and mask by 200 ms). Interestingly, their results hint at the possibility of some intervals *not* being effective: their Figure 9 shows the largest effect at the shortest and largest interval, and the interaction of congruency with interval gives a *p* value of .10. This is also the conclusion the authors adhere to, as they claim that “RTs were influenced at the earliest and latest SOAs” (Ostarek & Huettig, 2017, p. 504). However, in the absence of statistical evidence for differential effectiveness in the Ostarek study, we conclude, based on our results and that of three similar studies (Lupyan, Forder, Ostarek), that linguistic–visual interactions are effective over a wide range of intervals. The latter is in line with a recent MEG study showing that spoken words improved object category decoding between 200–500 ms after onset of the visual target, using a SOA (spoken word to visual target) that varied between 900 and 1,100 ms (Brandman et al., 2020).

In conclusion, we have shown that visual targets are propelled into awareness faster when accompanied by congruent compared to incongruent verbal linguistic labels, even when minimizing the possibility that labels were held active in working memory. As a cautionary note, we are adamant in stressing that we do not take our results to imply that verbal linguistic labels affect *unconscious* processing of the visual targets per se. The reason for this is that we line up with authors arguing that differences in b-CFS breakthrough times should not be taken, without additional evidence, to implicate differential unconscious processing (Gayet et al., 2014; Stein, 2019; Stein & Sterzer, 2014). Future studies could perhaps find out whether this difference in the time it takes to access awareness is the result of preactivation of congruent visual targets (as was implicated as a mechanism by Gayet, van Maanen, et al., 2016). According to this explanation, linguistic labels elevate the base activation level for matching visual representations (e.g., red). Such a hypothesis is not farfetched given that linguistic labels have been shown to activate neural representations at early sensory stages, as suggested by EEG data (Forder et al., 2017). Such a preactivation account could be similar to that proposed by Brandman et al. (2020) who suggested that words activate semantic representations which activate corresponding visual representations. Additionally, such an account would provide an explicit mechanism for the mounting evidence supporting the idea that cognition is grounded: hearing the word ‘red’ makes one spontaneously activate its experienced visual (and possibly gustatory, olfactory, and somatosensory) impression.

## Supplementary Information

ESM 1 (DOCX 72 kb)

## Footnotes

1 Note that Lupyan and Ward (2013) and Ostarek and Huettig (2017) used continuous flash suppression and Forder et al. (2016) used *breaking* continuous flash suppression, a notable issue we will return to further on.

2 As Stein (2019) puts it: ‘Some studies fixed presentation times but combined an accuracy-based with an RT-based task, […] Here, additional speeded response requirements could have interfered with perceptual sensitivity, such that differences in response criteria between conditions could inadvertently have had an effect on detection sensitivity.’

3 Although caution has to be taken when inferring *unconscious* processing of information preceding conscious access of information (an issue we return to in the General Discussion).

4 In the context of that study labelled a cuing effect.

5 This dual-mechanism model of visual masking was introduced by (Breitmeyer & Ögmen, 2006).

6 For example, Kanai et al. (2005) showed that transients can change the percept when viewing bistable stimuli. Such a change in percept could also occur by a noise burst.
