# Supplementary material for: Unpredictive linguistic verbal cues accelerate congruent visual targets into awareness in a breaking continuous flash suppression paradigm
Source: Atten Percept Psychophys. 2021 Mar 30;83(5):2102–12. doi: 10.3758/s13414-021-02297-y (PMC8213547; doi:10.3758/s13414-021-02297-y)
Supplement: Supplementary file 1 — (DOCX 72 kb) [file 13414_2021_2297_MOESM1_ESM.docx]

**Supplementary figures**


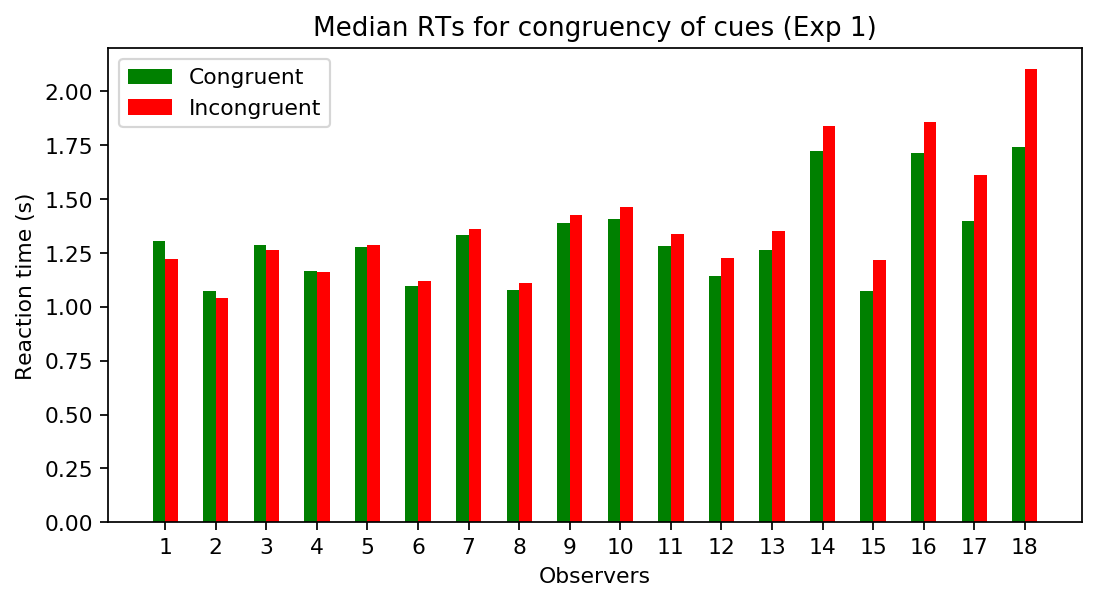


*Figure S1. Individual results for Experiment 1, per observer pooled over the timing of the auditory cue.*


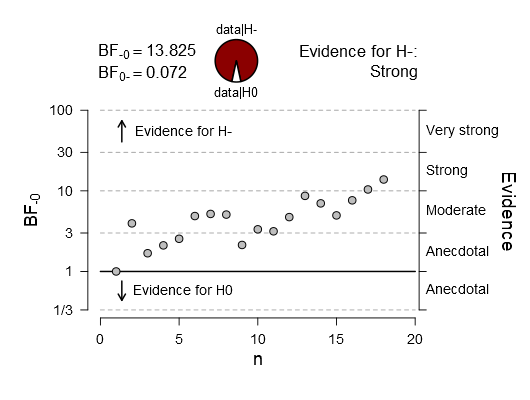


*Figure S2. Results of the Bayesian stopping rule in Experiment 1. Data collection was terminated after the Bayes Factor reached a factor of 10 or higher (or 1/10 or lower).*
